# Supplementary material for: Temporal Contrastive Learning through implicit non-equilibrium memory
Source: Nat Commun. 2025 Mar 4;16:2163. doi: 10.1038/s41467-025-57043-x (PMC11880436; doi:10.1038/s41467-025-57043-x)
Supplement: Supplementary file 1 — Supplementary Information [file 41467_2025_57043_MOESM1_ESM.pdf]

Supplemental Information: Temporal Contrastive Learning through implicit non-equilibrium memory

Martin J. Falk[1,+], Adam T. Strupp[1,+], Benjamin Scellier[2], Arvind Murugan[1]
[+]*denotes equal contribution*
[1]*Department of Physics, University of Chicago, Chicago, IL 60637 and*
[2]*Rain AI, San Francisco, CA 94110*
(Dated: February 24, 2025)

S1. KERNEL-BASED LEARNING RULES THAT APPROXIMATE CONTRASTIVE LEARNING

Our generalized Hebbian learning framework is based on weight updates of the form in main text Eq. 6, replicated here:

$$\Delta w_{ij} = \epsilon \int_0^{\tau_f+\tau_s} g\left(\int_{-\infty}^t K(t-t')s_{ij}(t')\,dt'\right)\,dt \ , \tag{S1}$$

with ϵ the learning rate. As discussed in main text Eq. 11, the temporal forcing from the synaptic current $s_{ij}(t)$ is an asymmetric sawtooth wave composed of two linear segments: a fast transition from the free value s_{ij}^{free} to the clamped value s_{ij}^{clamped} occurring over a timescale τ_f, and then a slow relaxation from clamped value s_{ij}^{clamped} back to free value s_{ij}^{free} over a timescale τ_s:

$$s_{ij}(t) = \begin{cases} \frac{At}{\tau_f} + \overline{s_{ij}} - \frac{A}{2} & t \le \tau_f \\ \overline{s_{ij}} + \frac{A}{2} - \frac{A(t-\tau_f)}{\tau_s} & \tau_f < t \le \tau_s \end{cases} \ , \tag{S2}$$

where $A = s_{ij}^{\text{clamped}} - s_{ij}^{\text{free}}$ and $\overline{s_{ij}} = \frac{1}{2}(s_{ij}^{\text{clamped}} + s_{ij}^{\text{free}})$. The nonlinearity g is also of the form discussed in main text Eq. 5, i.e. 0 below a threshold magnitude θ_g and linear elsewhere:

$$g(u) = \begin{cases} u & |u| \ge \theta_g \\ 0 & |u| < \theta_g \end{cases} \ . \tag{S3}$$

For the moment, we assume the kernel K to be an arbitrary function with a characteristic timescale τ_K.

In this section, we compute conditions on K, τ_K, τ_f, τ_s and θ_g such that our weight update in main text Eq. 6 approximates an ideal contrastive rule:

$$\Delta w_{ij} \approx \epsilon(s_{ij}^{\text{clamped}} - s_{ij}^{\text{free}}), \tag{S4}$$

with learning rate ϵ. We will find that the ideal contrastive rule can be approximated in the following regime:

1. $I = \int_0^\infty K(t)dt = 0$;
2. $\tau_K \ll \tau_f \ll \tau_s$.

We proceed in three steps. First, we will assume that $\tau_K \ll \tau_f$ and show that if $I = 0$, then convolution of linear s_{ij} with kernel K results in an output proportional to $\dot{s}_{ij}$, the time-derivative of s_{ij}. Second, we will show that $\tau_f \ll \tau_s$ in order to distinguish free-to-clamped and clamped-to-free transitions. Finally, we will empirically show that contrastive updates break down if $\tau_K \to \tau_f$ from above.

A. Zero-area kernels are required for contrastive updates

Our first goal is to find conditions for:

$$\int_{-\infty}^t K(t-t')s_{ij}(t')\,dt' \approx \frac{ds_{ij}(t)}{dt}. \tag{S5}$$

This is a prerequisite to reproducing contrastive updates such as main text Eq. 7. We will first assume that $\tau_K \ll \tau_f$ and hence that on the timescale of the integral in Eq. S5, $s_{ij}(t) = mt + b$ for some slope m and some y-intercept b.

Performing the integration on the LHS of Eq. S5 yields:

$$b\int_{-\infty}^t K(t-t')dt' + m\int_{-\infty}^t K(t-t')t'dt'. \tag{S6}$$

By u-substitution ($u = t - t'$) this is:

$$b\int_0^\infty K(u)du + m\int_0^\infty tK(u)du \ - \ m\int_0^\infty uK(u)du, \tag{S7}$$

which results in the following:

$$\int_{-\infty}^{t} K(t-t')s_{ij}(t')dt' = (mt+b)I - m(M_1^K), \tag{S8}$$

where (M_1^K) is the first moment of $K(t)$ around zero and I is the area under the kernel provided $K(t) = 0$ on $(-\infty, 0)$. Putting this together yields:

$$\int_{-\infty}^{t} K(t-t')s_{ij}(t')dt' = s_{ij}(t)I - m(M_1^K)\ , \tag{S9}$$

Here $s_{ij}(t)$ is the instantaneous signal value, M_1^K is the first moment of $K(x)$ around 0 and I is the total area under the kernel. In order for this integral to equal m, it is desirable that:

$$I = 0, \quad M_1^K = -1\ . \tag{S10}$$

Eq. S9 implies that if we choose kernels K with integrated area $I = 0$, then convolution of K with a linear synaptic current $s_{ij}(t)$ will extract values proportional to the slope m of the synaptic current. Further, we can choose the first moment of K such that the value of the convolution is equal to the slope m and not just proportional, though this is not strictly necessary.

B. Separation of slow and fast training timescales is optimal for contrastive updates

The next step is to investigate constraints on the nonlinearity threshold θ_g, τ_f, and τ_K.

In the previous section, we showed that if $I = 0$ and $\tau_K \ll \tau_f$, then we can approximate the convolution of kernel K with s_{ij} as being equal to $\dot{s}_{ij}$. In this limit, we can rewrite the weight update main text Eq. 6 as:

$$\epsilon^{-1}\Delta w_{ij} \ \approx \int_{0}^{\tau_f} g\left(\frac{ds_{ij}}{dt}\right)dt + \int_{\tau_f}^{\tau_f+\tau_s} g\left(\frac{ds_{ij}}{dt}\right)dt, \tag{S11}$$

with learning rate ϵ.

In order to reproduce contrastive updates, we require the first integral to not vanish over its entire range of integration, while the second integration should vanish uniformly. This implies that, for a synaptic current of amplitude $A = s_{ij}^{\text{clamped}} - s_{ij}^{\text{free}}$, fast timescale τ_f, and slow timescale τ_s, the nonlinearity threshold θ_g must satisfy:

$$\frac{A}{\tau_s} < \theta_g < \frac{A}{\tau_f}. \tag{S12}$$

If we require contrastive learning to occur over a range of amplitudes between A_{min} and A_{max} for fixed θ_g, then this condition written over all amplitudes becomes:

$$\frac{A_{max}}{\tau_s} < \theta_g < \frac{A_{min}}{\tau_f}. \tag{S13}$$

Saturating this bound yields:

$$\frac{\tau_s}{\tau_f} > \frac{A_{max}}{A_{min}},$$

implying that our protocol has optimal dynamic range of amplitude when $\tau_f \ll \tau_s$.

C. Kernel timescales must be faster than training timescales for contrastive updates

In deriving the conditions $I = 0$ and $\tau_f \ll \tau_s$ for successful contrastive updates, we assumed $\tau_K \ll \tau_f$. The latter inequality allowed us to approximate the instantaneous convolution of kernel K with synaptic protocol s_{ij} as convolution with a linear segment, ignoring the exact sawtooth functional form of s_{ij}.

We additionally show that $\tau_K \ll \tau_f$ is required empirically. For fixed τ_K and τ_s, we varied τ_f and computed the weight update computed using main text Eq. 6, using a kernel of a form discussed in Eq. S35. We compared this to the ideal contrastive update, which is equal to the amplitude of the protocol s_{ij}. We find that $\tau_K \ll \tau_f$ in order for the weight update to approach the ideal contrastive update (Fig. S1).

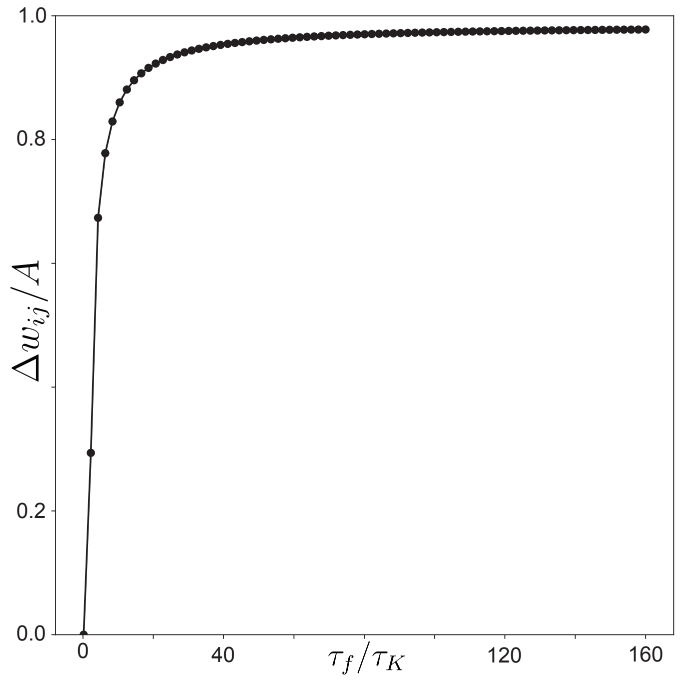

FIG. S1. **Finite-time derivative breakdown for short signals.** Simulation of kernel convolution ability to capture amplitude of a signal with short timescale. The x-axis shows ratio between timescale of the signal upswing τ_f and the memory kernel τ_K. The y-axis gives the ratio of weight update (as given in main text Eq. 6) to the actual amplitude of the signal. A ratio of 1 reflects the kernel convolution approximating well the finite time derivative. At high τ_f, the ratio approaches 1 as desired. At low τ_f, it becomes unreliable. Convolutions were run numerically for 80 different values of $\tau_f \in (0, 8)$, convolving a signal with amplitude 20, with $dt = 1e - 4$. Here we fix $\tau_s = 20$. The kernel used was of the form given by Eq. S35 and had net 0 area. The nonlinearity threshold θ_g was chosen to be the average of the lowest u_{ij} value on the upswing and the highest u_{ij} value on the downswing.

S2. RELATED WORK

In this section, we review existing methods to implement contrastive rules, and we show how our proposal compares with these methods.

a. Storing the states. The contrastive learning rule

$$\Delta w_{ij} = \epsilon \left(s_{ij}^{\text{clamped}} - s_{ij}^{\text{free}} \right) \tag{S14}$$

requires a comparison between free and nudge states, but the network cannot be in both states simultaneously. The first option, used in the experiments of Yi *et al.* [1] on memristor crossbar, and Laydevant *et al.* [2] on D-wave's Ising machine, is to store the states using an external memory.

b. Coupled learning. In their experimental realizations of contrastive learning on resistor networks, Dillavou *et al.* [3, 4] used two copies of the network, one for each state. Wycoff *et al.* [5] demonstrated in a resistor network that weights may be updated asynchronously, but they still require contrasting two states.

c. Switching between Hebbian and anti-Hebbian updates. To avoid storing states or using two networks, another solution consists in performing two updates rather than one - one update for each of the two states (free and nudge).

1. **Free phase.** Allow the network to reach the free state and we perform an 'anti-Hebbian' weight update:

$$\Delta w_{ij} = -\epsilon s_{ij}^{\text{free}}. \tag{S15}$$

2. **Clamped phase.** Allow the network to reach the clamped state and we perform a 'Hebbian' weight update:

$$\Delta w_{ij} = \epsilon s_{ij}^{\text{clamped}}. \tag{S16}$$

Another closely related method, proposed by Williams *et al.* [6] is, for each training example, to perform only one of the two updates, drawn at random. This method provides an unbiased, but high variance, estimator of the weight

gradient. Williams *et al.* [6] also explore schemes where the weight update is activated during the entire phase, and with phases of variable duration (before reaching steady state). However, these methods requires two different weight update mechanisms for each of the two phases: they require switching between Hebbian and anti-Hebbian updates.

d. Continual EP. Ernoult *et al.* [7] rewrite the contrastive learning rule as

$$s_{ij}^{\text{clamped}} - s_{ij}^{\text{free}} = \int_{\text{free}}^{\text{clamped}} \frac{ds_{ij}}{dt} dt, \tag{S17}$$

along any trajectory driving the system from free state to clamped state. Using this formulation of the learning rule, they propose an implementation of contrastive learning with continual weight updates in the clamped phase. The algorithm proceeds as follows:

1. **Free phase.** Let the network to reach the free state, while deactivating any weight update, i.e.

$$\frac{dw_{ij}}{dt} = 0. \tag{S18}$$

2. **Clamped phase.** Starting from the free state, let the network reach the clamped state, while turning on the weight update

$$\frac{dw_{ij}}{dt} = \epsilon \frac{ds_{ij}}{dt} \tag{S19}$$

along the trajectory from free state to clamped state.

In this approach again, one caveat is that it requires switching between two distinct weight update mechanisms. Our algorithm overcomes this hurdle.

e. Using oscillations. Baldi and Pineda [8] proposed to use a periodic nudging signal, e.g. a sinusoidal nudging signal with amplitude β and frequency w. The weights are then updated using the system's response to this periodic nudging signal, denoted as $s(t)$, as

$$\Delta w_{ij} = \epsilon \int_0^{2\pi/\omega} \sin(\omega t) s_{ij}(t) dt. \tag{S20}$$

where $T = 2\pi/\omega$ is the period. Similar to other schemes, one caveat of this learning rule is that it requires being modulated by a time-varying factor $\sin(\omega t)$. [9] reused the idea of oscillations but, instead of the learning rule of Eq. (S20), they showed that in resistor, flow and elastic networks, the weight update for w_{ij} can be performed using the mean and amplitude of $s_{ij}(t)$. However, it remains unclear how the mean and amplitude of the signal can be easily extracted.

f. Connection with STDP. Spike-timing-dependent plasticity (STDP) (Bi and Poo [10]) can be formulated as a learning rule for asymmetric networks where a synapse distinguishes between the time of pre-synaptic spikes t_k and the time of postsynaptic spikes t_j:

$$\Delta w_{ij} = f(t_j - t_k) \tag{S21}$$

where f has a positive lobe for positive arguments and a negative lobe for negative arguments, and approximately vanishes outside a pairing range $[-\tau, \tau]$.

A large body of work has interrogated the equivalence of spike- and rate-based formulations of STDP[11, 12]. In particular, Xie and Seung [13] considered additive STDP updates for δ-function timeseries $s_j(t)$, written as:

$$\Delta w_{ij} = \int_0^T dt_k \int_{-\infty}^{\infty} dt_j \; f(t_j - t_k) s_j(t_j) s_k(t_k) \tag{S22}$$

over a time period $[0, T]$. They showed that Eq. S22 can be approximated as rate-based formulations depending on firing rates $\nu_j(t)$ when rates of spiking vary slowly compared to the pairing range:

$$\Delta w_{ij} = \int_0^T dt \; [\beta_0 \nu_j(t) + \beta_1 \dot{\nu_j}(t)] \nu_k(t) \tag{S23}$$

where β_0 is the integral of f over the pairing range, and β_1 is the first moment of f over the pairing range.

In the case where $\beta_0 = 0$ and $\beta_1 > 0$, Eq. S23 can be interpreted as the asymmetric version of the nudge phase update of Continual EP (Eq. S19):

$$\frac{dw_{ij}}{dt} = \beta_1 \dot{\nu_j}(t)\nu_k(t). \tag{S24}$$

Martin *et al.* [14] proposed a spiking version of contrastive learning to train spiking networks with bidirectional weights. Similar to the Continual EP approach, in the free phase, no weight update occurs, and in the nudge phase, weights are updated through spikes based on a learning rule similar to Eq. (S19). They show that a version of STDP emerges from this learning rule.

g. Competitive Hebbian updates. In a directional context with pre-synaptic neuron i and postsynaptic neuron j, Journé *et al.* [15] consider weight updates of the form:

$$\Delta w_{ij} = \epsilon y_j (x_i - u_j w_{ij}) \tag{S25}$$

where x_i is the activation of neuron i and u_j is the weighted input to neuron j; y_j is a winner-take-all factor across all K neurons in a given layer, parameterized by a factor b:

$$y_j = \frac{b^{u_k}}{\sum_{l=1}^{K} b^{u_l}}. \tag{S26}$$

This method of learning is well-suited to a neuronal context. However, implementing it in less internally complex physical and biological systems would be a non-trivial task, requiring e.g. the engineering of equivalents to pre- and post-synaptic neurons, as well as the computation of the y_j Boltzmann factor. The latter could be accomplished potentially through lateral inhibition within layers[16], at the cost of making architectural assumptions that would narrow the range of natural settings the method could be implemented in.

h. Agnostic Equilibrium Propagation (AEP). Scellier *et al.* [17] introduced another dynamical version of EP called AEP, in which the trainable weights (w) evolve under physical dynamics to optimize the cost function. In AEP, both the weights (w) and the state variables (η) minimize the system's energy. The weights are strongly coupled to *control variables* (v) by a coupling term $U(v, w)$, so the total energy is $U(v, w) + E(w, \eta)$. The control variables can be in either of two states: clamped, or maintain homeostatic control over w.

At each training step t with current weights $w^{(t)}$, AEP proceeds as follows. In the free phase, an input is presented, and w and η jointly minimize the energy function, while $v^{(t)}$ performs homeostatic control, keeping w at its value $w^{(t)}$, i.e.

$$(w^{(t)}, \eta^{\text{free}}) = \underset{(w,\eta)}{\arg\min} \left[U(v^{(t)}, w) + E(w, \eta) \right]. \tag{S27}$$

In the clamped phase, the control $v^{(t)}$ is held fixed, and a nudging term $\beta C(\eta)$ is added to the system's energy (where C is the cost function to optimize and β is the nudging strength). The system settles to

$$(w^{(t+1)}, \eta^{\text{clamped}}) = \underset{(w,\eta)}{\arg\min} \left[U(v^{(t)}, w) + E(w, \eta) + \beta C(\eta) \right]. \tag{S28}$$

Scellier *et al.* [17] show that the weight change $w^{(t+1)} - w^{(t)}$ between free and clamped phases approximates one step of gradient descent on C.

In contrast, our Temporal Contrastive Learning (TCL) approach neither relies on homeostatic control nor requires w to minimize the system's energy.

i. Temporal Contrastive Learning (our method). In contrast to these other methods, we propose a mechanism based on integral feedback that uses a unique weight update rule (a unique learning mode) for both phases. Our proposal is related to, but different from, Continual EP, as we explain next.

The central idea in our approach is to introduce a kernel-based non-equilibrium memory $u_{ij}(t)$ for each variable s_{ij}, to approximate the time derivative $\frac{ds_{ij}}{dt}$,

$$u_{ij}(t) := \int_{-\infty}^{t} K(t - t') s_{ij}(t') dt' \approx \frac{ds_{ij}}{dt}. \tag{S29}$$

Intuitively, the memory kernel $K(t-t')$ approximates the derivative of the delta function (Dirac function), in the sense of distribution theory. We then use this non-equilibrium memory to drive the weight updates similar to Eq. (S19),

$$\frac{dw_{ij}}{dt} = g(u_{ij}(t)). \tag{S30}$$

However, we also need any weight update to vanish on the trajectory from the nudge state to the free state, as in Eq. (S18). To achieve this, we introduce two distinct time scales, τ_{clamped} and τ_{free} with $\tau_{\text{free}} \gg \tau_{\text{clamped}}$, so it takes a time τ_{clamped} to go from free state to nudge state, and a time τ_{free} to go from nudge state back to free state. Moreover, we use the nonlinear function

$$g(u) := \begin{cases} u & |u| \geq \theta_g \\ 0 & |u| < \theta_g \end{cases} , \tag{S31}$$

to suppress inputs below the characteristic threshold θ_g. The network is always in the same learning mode, whether in free or nudge phase. We break the symmetry between free and nudge phase, by using a fast change in the nudge phase (small τ_{clamped}) and subsequent slower relaxation in the free phase ($\tau_{\text{free}} \gg \tau_{\text{clamped}}$), so that the nonlinearity g suppresses weight updates that might occur during the slow nudge-to-free transition.

S3. QUANTITATIVE TIME AND ENERGY COMPARISON OF TCL VS OTHER CONTRASTIVE METHODS

Our work here on TCL is a proposal for how to implement contrastive learning physically in a fully analog manner; other related proposals (e.g., FF and other CL methods) typically involve digital processing to store two states. Consequently, the primary advantage of our method compared to contrastive algorithms is that it eliminates the need to store and fetch an explicit recording of free and clamped states (or, e.g. positive and negative states in the Forward-Forward algorithm[18]). TCL accomplishes this by embedding the difference between free and clamped states into a dynamical signal which is generated by integral feedback. This integral feedback mechanism allows for the accurate computation of contrastive weight updates (main text Eq. 6) at the expense of time and energy.

For TCL, the time cost of computing main text Eq. 6 is set by the dynamic range to which we want to be able to measure the contrastive difference. For example, if we want to be able to distinguish differences between free and clamped states by a ratio of 10^{-2}, this would require $\sim 10^3\tau_K$, where τ_K is the timescale of the physical processes generating integral feedback in the analog system (Fig. S1). Similarly, there is a cost associated with the energy dissipation rate σ of running integral feedback in each synapse, which takes about $\sim 5 - 10kT$ in order to achieve 10^{-2} relative error to the explicit contrastive update main text Eq. 6 (Fig. 6). The total energy cost of running the network per cycle therefore would be $\sim 10kTN_{synapse}(\tau_f + \tau_s)$, with the number of synapses $N_{synapse}$ scaling with the number of nodes N or N^2 depending on network connectivity.

For explicitly contrastive implementations, one time cost of computing main text Eq. 6 is instead the time of storing each state and retrieving it to perform the difference operation. For memory stored on a digital device locally at each node, this is a constant time, τ_{fetch}. For fully digital implementations (e.g. simulations of EP methods on a computer) this time is extensive in N, i.e. $N\tau_{fetch}$. This is in addition to the time cost of relaxation of the physical state of the system in response to alternating between clamping and releasing the output nodes, which we label τ_{relax}. Therefore, to simulate, for example Equilibrium Propagation[19] on a computer would naively take a time $2\tau_{relax} + N\tau_{fetch}$ per cycle. In a twinned circuit device as constructed in Refs. [3, 20], the time per cycle would be $\tau_{relax} + \tau_{fetch}$.

Energy costs associated with explicit contrastive algorithms arise from the need to continually write and erase the state of the system. Assuming that memory is written at each of the N nodes and is required to an accuracy of n bits, a minimum energy required for contrastive algorithms is $NnkT\ln 2$ following the Landauer estimate[21].

We note that it is hard to generate fair, apples-to-apples comparisons of energy and time costs between TCL and other contrastive algorithms because we do not yet have concrete analog proposals for other contrastive algorithms; some key aspect of those algorithms require digital operations[3, 22] such as using digital memory to actually compute main text Eq. 6. Nevertheless, we have presented quantitative costs for the TCL proposal to allow for easy comparison to any new analog contrastive learning proposals as they become available.

S4. TRAINING MULTI-SYNAPSE NETWORKS

Here, we describe in greater detail how we trained a neural network to classify MNIST digits using our proposed contrastive learning framework. We adapt code from Ref. [19], originally used to classify MNIST digits using the Equilibrium Propagation algorithm. We train on a 10000-element subset of MNIST with all digits, with holdout test set of size 2000. The architecture of the network trained has 784 input nodes, 500 hidden nodes, and 10 output nodes, each with a state x. Each layer is fully connected to the subsequent layer, with no intra-layer connections and no skip connections.

Dynamics of the network during inference minimize the energy functional:

$$E(x) = \frac{1}{2}\Sigma_i x_i^2 - \frac{1}{2}\Sigma_{i,j} w_{ij}\eta_i\eta_j - \Sigma_i b_i\eta_i, \tag{S32}$$

where b_i is the bias of node i, w_{ij} is the weight of the synapse connecting nodes i and j, and the indices i, j run over the node indices of all layers (input, hidden, and output). Additionally, η is a non-linear activation function, in this case $\eta(x) = \text{clip}(x, 0, 1)$. During the training of the network, the network instead minimizes:

$$F(x;t) = E(x) + \frac{\beta(t)}{2}\Sigma_{o=0}^{9}(x_o - v_o^{\text{label}})^2. \tag{S33}$$

Here, v_o^{label} is the one-hot encoding of the desired MNIST output class, and the x_o are the states of the 10 neurons in the output layer. β is a clamping parameter which varies in time as a sawtooth function.

During both training and inference, the goal is to minimize the energy F with respect to the neuronal state variables x. A persistent particle trick was employed to keep track of the free phase values for x for a given MNIST entry, to cut down on the amount of time needed to relax to the energy minimum when changing between MNIST entries. Weights are initialized by the same Glorot-Bengio initialization[23] as in Ref. [19], but divided by a factor of 2. See Ref. [19] Secs. 5.1, 5.2 for further details.

In the original implementation of Ref. [19], updates to w_{ij} and b_i are proportional to the difference between the state variables in the free state and a single clamped state. Our goal is instead to subject the neural network to a time-varying sawtooth protocol $\beta(t)$ (e.g. main text Eq. 11) with a fast upswing from free to clamped states, and a slow relaxation back to free.

In order to approximate this protocol, we move the neural network in small discrete steps from a state with clamping parameter $\beta = 0$ to a state with $\beta = \beta_{max}$, and then back down to the $\beta = 0$ state. In the numerical experiment reported in Fig. 3, we choose $|\beta_{max}| = .5$, with the sign of β_{max} chosen randomly, following Ref. [19] Section 5.2. We discretize both the upswing and downswing of the β ramp into 9 pieces each, for 20 timepoints total. We follow the heuristic set in Ref. [19] and allow the system to initially relax for 20 iterations in the free phase, and then for 4 iterations at each subsequent point in the β ramp. Each iteration updates the system following gradient descent of F with respect to the states x_i, with a step size of .5. At each point in the ramp, we record the gradient of the energy F with respect to w_{ij} and b_i, which are respectively the synaptic current $\eta(x_i)\eta(x_j)$ and the activation $\eta(x_i)$. This will allow us to compute the weight update main text Eq. 6 downstream.

Note that this procedure of needing to record the timeseries of x as β is changed is a proxy for the feedback dynamics that we are proposing would be naturally implemented in a physical system.

To map the system behavior onto the timeseries the neural network would experience if forced by an asymmetric sawtooth protocol as in main text Eq. 11, we use the SciPy interp1d function with default parameters. We interpolate the values of x onto a timeseries of total time 1, $dt = .01$, and a fast upswing time $\tau_f = .1$ and a slow relaxation time $\tau_s = .9$.

We are now in a position to compute weight updates for our neural network following main text Eq. 6. We construct a kernel:

$$K(t) = \frac{1}{3}\sin(20\pi t) \tag{S34}$$

with $0 < t < .1$ and a time resolution $dt = .01$. The nonlinear threshold function g has a layer-dependent nonlinearity; input-to-hidden layer w_{ij} threshold is 1.2×10^{-6}, hidden layer b_i threshold is 10^{-5}, hidden-to-output layer w_{ij} threshold is 2×10^{-5}, and output layer b_i threshold is 2×10^{-4}. Input layer b_i are not modified during training.

We perform the convolution of our interpolated timeseries of synaptic currents and activations with kernel K (the inner integral of main text Eq. 6) with the SciPy convolve1d function in the "wrap" mode for periodic signals. We divide the result by 100 to account for the time resolution of the signal. We then pass the convolution through g and integrate the result using the SciPy trapz function with default parameters. This result is Δw_{ij} from main text Eq. 6.

Next, we perform change existing weights according to $\frac{10^4\epsilon}{2\beta_{max}}\Delta w_{ij}$. The ϵ are layer-dependent learning rates, set to .1 for hidden layer associated weights and .05 for output layer associated weights. For our neural network, we added the factor of 10^4 empirically to speed convergence. We perform this procedure of weight updates over minibatches of size 20, with 500 minibatches in the training set and 100 in the testing set.

Finally, we need a method to evaluate the performance of our trained network on predicting MNIST labels for unseen data. When evaluating the network on the testing set, the network classification of the MNIST image input is the argument of the neuron with the maximal output. If this argument does not match the label of the MNIST input, then the network has misclassified the input. In Fig. 3B, we report the average classification error after each epoch of training, where a single epoch consists of training the network on all 500 training set minibatches and evaluating the network on all 100 testing set minibatches.

S5. KERNEL COEFFICIENTS USED IN ASSESSING SPEED-ACCURACY TRADEOFF

In order to assess limitations of our proposed contrastive learning framework, we performed a detailed analysis of learning dynamics in a single synapse. These results, reported in Fig. 4, made use of kernels that have been studied before in Ref. [24]. These kernels have the functional form:

$$K(t-t') \;=\; \alpha e^{-\tau_K^{-1}(t-t')}((t-t')-\lambda(t-t')^2). \tag{S35}$$

Here we briefly detail our choices for the coefficients α, τ_K, λ for such kernels when used in our single-synapse analysis.

First, we fix $\tau_K = \frac{1}{20}$ for quick decay, which simplified numerical calculation. Second, following Section S1, we require $I = \int_0^\infty K(t)dt = 0$. Third, again following Section S1, our calculations are simplified if the first moment $M_1^K = -1$.

The latter two requirements allow us to fix α and λ as:

$$\alpha = \tau_K^{-3} \;\;, \; \lambda = \frac{1}{2\tau_K}. \tag{S36}$$

Numerically, the memory kernel is created with unit length and timestep $dt = 1e-4$.

S6. WEIGHT UPDATES AND OPTIMAL THRESHOLDS FOR KERNELS WITH NON-ZERO AREA

Our single synapse analysis was first performed in an ideal limit, assuming kernels with zero area. In Fig. 5, we generalized those results to non-ideal scenarios where kernels have non-zero integrated area.

In this section we discuss how weight updates are modified when we leave the integrated area $I = \int_0^\infty K(t)dt = 0$ regime, which is ideal for contrastive updates (see Section S1 for the $I = 0$ case). We also address the question of choosing the threshold value θ_g for the nonlinearity g in the $I \neq 0$ regime. We provide a brief note on constructing kernels with a controlled amount of integrated area I.

A. Weight updates for kernels with non-zero area

In Eq. S9, we derived the relation:

$$\int_{-\infty}^{t} K(t-t')s_{ij}(t')\,dt' = s_{ij}(t)I - m(M_1^K), \tag{S37}$$

in the limit as $\tau_K \ll \tau_f$ for an arbitarry kernel K and a linear $s_{ij}(t) = mt + b$. M_1^K is the first moment of the kernel.

Evaluated on the fast rise of time τ_f from free to clamped states, $m = \frac{(s_{ij}^{\text{clamped}} - s_{ij}^{\text{free}})}{\tau_f}$. If we assume $M_0^K = -1$, but $I \neq 0$, we have the following:

$$\int_{-\infty}^{t} K(t-t')s_{ij}(t')dt' \approx \frac{(s_{ij}^{\text{clamped}} - s_{ij}^{\text{free}})}{\tau_f} + Is_{ij}(t). \tag{S38}$$

Then integrated over a cycle (assuming we can always threshold the slow relaxation from clamped to free out), this yields for the weight update:

$$\int_0^{\tau_f}\int_{-\infty}^{t} K(t-t')s_{ij}(t')dt'dt = (s_{ij}^{\text{clamped}} - s_{ij}^{\text{free}}) + I\tau_f\overline{s_{ij}}, \tag{S39}$$

where $\overline{s_{ij}} = (s_{ij}^{\text{clamped}} + s_{ij}^{\text{free}})/2$. Thus an offset of $I\tau_f\overline{s_{ij}}$ is added to the desired weight update (Fig. 5B).

B. Choosing optimal thresholds for kernels non-zero area

Next, we discuss an optimal choice for θ_g when $I \neq 0$.

The sawtooth wave used as the signal here takes to following form, as replicated from main text Eq. 11:

$$s_{ij}(t) = \begin{cases} \frac{At}{\tau_f} + \overline{s_{ij}} - \frac{A}{2} & t \leq \tau_f \\ \overline{s_{ij}} + \frac{A}{2} - \frac{A(t-\tau_f)}{\tau_s} & \tau_f \leq t \leq \tau_s \end{cases} . \tag{S40}$$

The value of the convolution measured in Eq. S9 takes on two distinct forms on the two sections of the sawtooth wave. Plugging $s_{ij}(t)$ from main text Eq. 11 into Eq. S9 we have:

$$u_{ij}(t) = \begin{cases} \frac{A}{\tau_f} + I(\frac{At}{\tau_f} + \overline{s_{ij}} - \frac{A}{2}) & t \leq \tau_f \\ -\frac{A}{\tau_s} + I(\overline{s_{ij}} + \frac{A}{2} - \frac{A(t-\tau_f)}{\tau_s}) & \tau_f \leq t \leq \tau_s \end{cases} . \tag{S41}$$

Since the performance of a kernel relies on its ability to capture the difference between the two sections of the sawtooth signal, one sufficient condition for a good protocol is the following:

$$\min \left(\int_{-\infty}^{t} K(t-t')s_{ij}(t')dt' \text{ on } (0, \tau_f) \right) > \theta_g \tag{S42}$$

$$\theta_g > \max \left(\int_{-\infty}^{t} K(t-t')s_{ij}(t')dt' \text{ on } (\tau_f, \tau_s) \right) . \tag{S43}$$

This condition ensures the values that are desired are all above threshold, while the values that should be suppressed (those on the slow relaxation timescale), are all thresholded out. It is a stronger condition than may be absolutely necessary for acceptable performance. This condition enforced onto $\int_{-\infty}^{t} K(t-t')s_{ij}(t')dt'$ as represented in Eq. S41 implies:

$$\frac{A}{\tau_f} + I(\overline{s_{ij}} - \frac{A}{2}) > -\frac{A}{\tau_s} + I(\overline{s_{ij}} + \frac{A}{2}) \ , \tag{S44}$$

assuming both sides are positive. This assumption, in the limit as $A \ll \overline{s_{ij}}$ requires I and $\overline{s_{ij}}$ to be the same sign.

In the limit as $A \gg \overline{s_{ij}}$, it requires:

$$\frac{2}{\tau_s} < I < \frac{2}{\tau_f}. \tag{S45}$$

The condition itself implies:

$$I < \frac{1}{\tau_f} + \frac{1}{\tau_s}. \tag{S46}$$

Combined, this implies:

$$\frac{2}{\tau_s} < I < \frac{1}{\tau_f} + \frac{1}{\tau_s}. \tag{S47}$$

This defines the tradeoff between timescales and kernel area that, if satisfied, guarantees the existence of a θ_g which distinguishes between the two sections of the sawtooth. In this work, we choose θ_g as the maximum of the second portion of convolution values:

$$\theta_g = -\frac{A_{max}}{\tau_s} + I(\overline{s_{ij}} + \frac{A_{max}}{2}). \tag{S48}$$

Per Eq. S47, this choice of θ_g is valid in regimes where I is sufficiently large to make the term involving I in Eq. S48 dominate. This ensures that the expression S48 is the absolute value of the most extreme value of u_{ij} on the downswing (i.e. the downswing is entirely thresholded out by g). This "adaptive" choice of θ_g is tuned to the maximum signal amplitude within an otherwise fixed protocol. It guarantees that the downswing of the sawtooth is thresholded out for all values of $A < A_{max}$, while for some values, portions of the upswing are above threshold. The inequality in Eq. S44 guarantees that the entire upswing is above threshold when $A = A_{max}$. However, this choice of θ_g leads to good performance even when inequality S44 isn't strictly met. This choice of θ_g is used in our simulations whenever I is non-negligible.

C. Coefficients for kernels with non-zero area

Here, we provide a brief note for how to construct kernels used in the Fig. 5 analysis, which is done for kernels with $I \neq 0$. In particular, the functional form of the kernels we chose is given by Eq. S35 but with $I \neq 0$. The following parameter constraints provide the desired integrated area I while maintaining the first moment $M_K^1 = -1$:

$$K(t-t') \ = \ \alpha e^{-\tau_K^{-1}(t-t')}((t-t') - \lambda(t-t')^2) \tag{S49}$$

$$\alpha = 3I\tau_K^{-2} + \tau_K^{-3} \tag{S50}$$

$$\lambda = \frac{\tau_K^{-2} + 2I\tau_K^{-1}}{6I + 2\tau_K^{-1}}. \tag{S51}$$

These parameter choices reduce to those given in Eq. S36 when $I = 0$.

S7. PROTOCOL EVALUATION METRICS

Below we outline two metrics that we used to evaluate the performance of our proposed learning protocol across Figs. 4-6.

Both metrics are derived from a plot of the computed weight update value Δw_{ij} and the sawtooth amplitude A as shown in Fig. 4B; a "weight update curve." This graph is created by calculating Δw_{ij} for signals over a range of amplitudes and recording pairs $(A, \Delta w_{ij})$ for 100 values of A between 0 and A_{max} using the linspace function from NumPy.

There are two main features that we extract from the weight update curve: offset and dynamic range. To calculate these values in the limit of low I, we use the PiecewiseLinFit function from the pwlf library to section the response curve into three linear segments. The offset is calculated as the y-intercept of the extension of the rightmost linear segment using the intercepts function from the same library. If the rightmost linear segment has a slope between .75 and 1.25 (a proxy for approximately a linear response with slope = 1) calculated with the slopes function from the same library, then A_{min} is defined as the left edge of this segment, calculated using the breaks function from pwlf.

This formulation is motivated by the observation that generated response curves had three regions. At low A, there is no response, then response spikes sharply as the threshold is passed, and then approximately linearly grows. The left edge of this segment therefore is a proxy for the range of approximately linear response. The offset is a proxy for the impact on background signal value which shifts this segment up or down. We use dynamic range = $\frac{A_{max}}{A_{min}}$ as the most explanatory measure.

S8. KERNELS BASED ON MARKOV STATE MODELS

In addition to investigating the effect of memory kernels described at a phenomenological level, we also investigated kernels derived from microscopic statistical mechanics models. Building off work by Ref. [25], who constructed a Markov state model for *E. coli* chemoreceptor adaptation, we used the model to construct kernels and investigated how varying the parameters in the network affected the kernels produced. These kernels allowed us to interrogate the relationship between dissipation and performance in our proposed contrastive learning framework (Fig. 6).

First we create a Markov chain with 10 nodes, as in Ref. [25]. The Markov chain is laid out as a 2 x 5 grid where nodes are connected only to their neighbors on the square grid, resembling a ladder. The occupancies of each node as well as the transition rates between nodes are stored in lists.

The evolution of a Markov chain is governed by the master equation given as:

$$\frac{d\vec{p}}{dt} = \boldsymbol{R}\vec{p}\,. \tag{S52}$$

Here $\vec{p}$ is a vector containing the occupancies of each node, while $\boldsymbol{R}$ is the transition rate matrix, and $\boldsymbol{r}_{ij}$ is the transition rate from node j to node i. Due to the connectivity of the network shown in Fig. S2, most of the rates are 0 and the rest are defined by a small number of parameters and relations given below. The horizontal rates in the direction of clockwise circulation (rightward on the top level and leftward on the bottom), are modulated by a parameter γ which controls the dissipation required for non-equilibrium circular flow:

$$r_{clockwise} = \gamma r_{counterclockwise}\ . \tag{S53}$$

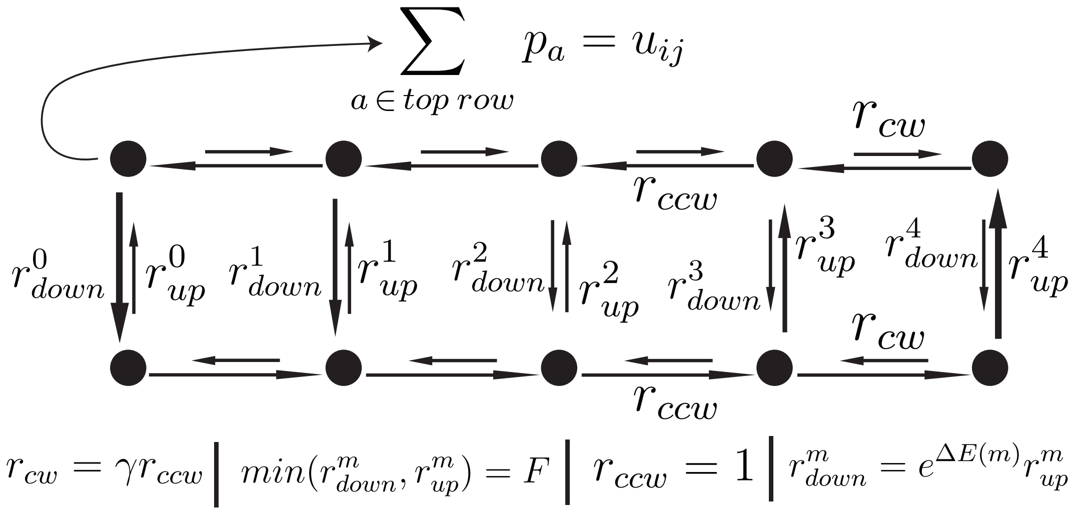

FIG. S2. **Structure of kernel-generating Markov state model.** The adaptive dynamics within each synapse are modeled with a 10-state Markov state model laid out as a 2×5 grid. The horizontal rates (r_{cw}, r_{ccw}) are parameterized by γ. The vertical rates (r^m_{up}, r^m_{down}) are parametrized by F and $\Delta E(s_{ij}, m)$. The sum of top row occupancies p_a is identified with $u_{ij} = \int_{-\infty}^{t} K(t-t') s_{ij}\, dt'$. The time derivative of u_{ij} then defines the memory kernel: $\frac{d}{dt} u_{ij}(t) = K(t)$.

Clockwise rates are fixed to the value 1 in this work for simplicity. There are 5 sets of vertical rates, r^n_{up} and r^n_{down} where m is an integer between 0 and 4 representing the horizontal position. There is an energy difference ΔE defined for each m given as:

$$\Delta E = 2(1-m) + \ln\left(\frac{1 + (\frac{s_{ij}}{18.2})}{1 + (\frac{s_{ij}}{3000})}\right) \,. \tag{S54}$$

Here s_{ij} is the instantaneous signal value. Following the model in Ref. [25], the vertical rates are a function of ΔE:

$$r^m_{down} = e^{\Delta E} r^m_{up} \quad , \quad min(r^m_{down}, r^m_{up}) = F, \tag{S55}$$

where F is a constant that governs the ratio between vertical and horizontal transition rates. It is default set to F = 25. A transition matrix can be constructed for any choice of rates defined above.

The process of finding the memory kernel requires bringing the Markov chain to a steady state and then perturbing it. The null_space function from the SciPy library linalg is used to get the null space of the transition matrix, which is the steady state of the Markov chain. A short time-series of occupancy values are captured for the Markov chain sitting in the steady state.

Time evolution of the Markov chain uses the odeint function from the SciPy integrate library to get a time-series of occupancy values. The argument "derivfun" is set to a function that implements the dynamical rule in Eq. S52. The argument "y0" is set to the null-space of the Markov chain.

Starting in the steady state, the transition matrix of the Markov chain is then changed by perturbing the variable s from s = 0 to some higher value in a step function form. This causes flow of occupancy in the Markov chain before settling into a new steady state. As this evolution occurs, the occupancy u_{ij} of the top level of the Markov chain is summed and saved as a time-series of length 5 with density $dt = 5 \times 10^{-4}$ for a total of 10,000 values stored in a 1-dimensional array.

The kernel is then defined as the derivative of the step function response (the graph of top level occupancy over time after step function perturbation of s_{ij}). The top level occupancy is identified with the signal u_{ij}:

$$\sum_{a \in top\,row} p_a \equiv u_{ij}. \tag{S56}$$

Then u_{ij} is defined as usual:

$$u_{ij}(t) = \int_{-\infty}^{t} K(t-t') s_{ij}(t') dt' \tag{S57}$$

We use the following general result:

$$\frac{d}{dx}\left(\int_{-\infty}^{x} f(x-t')g(t')dt'\right) = \int_{-\infty}^{x} f(x-t')\frac{dg}{dx}(t')dt' \tag{S58}$$

We substitute into Eq. S57.

$$\frac{d}{dt}u_{ij}(t) = \int_{-\infty}^{t} K(t-t')\frac{ds_{ij}}{dt}(t')dt' \tag{S59}$$

For s_{ij} chosen to be a step function S, we have

$$\int_{-\infty}^{t} K(t-t')\frac{dS}{dt}(t')dt' = \int_{-\infty}^{t} K(t-t')\lambda(t-t')dt' = K(t). \tag{S60}$$

We thus arrive at our process of extracting the kernel K from each Markov chain:

$$\frac{d}{dt}\left(\sum_{a \in top\ row} p_a\right) = K(t) \tag{S61}$$

The derivative function from the SciPy misc library is used to calculate this derivative, passing "order = 3". Each kernel K is then normalized to have first moment M_1^K= -1.

One other relevant measure that is associated with a Markov chain, is its dissipation, defined as:

$$\sigma = \sum_{i>j}(r_{ij}p_j - r_{ji}p_i)\ln\left(\frac{r_{ij}p_j}{r_{ij}p_j}\right) . \tag{S62}$$

This quantity is calculated in the steady state, and over time during the evolution of the Markov chain.

S9. IMPLEMENTATION DETAILS FOR FIGS. 4-6

A. I = 0 Data

The data for Fig. 4C was generated in a parameter regime with $I = \overline{s_{ij}} = 0$. NumPy.random.randint was used to randomly choose τ_f in the range (0.1, 5) and τ_s in the range (0.2, 50). The *E. coli* kernel is constructed with specified area $I = 0$. Then the weight update curve is generated with the inputs $A_{max} = 0, \overline{s_{ij}} = 0$ and dynamic range and offset are calculated from that graph. Then τ_f, τ_s, A_{min}, and offset are saved into an array. We run 100 parallel batches of this calculation, with each batch generating 10 data points for a total of 1000 points of data. All colorbars used in this work are created with the Matplotlib colors library.

B. I = 0.05, $\overline{s_{ij}} = 25$ Data

The data for Fig. 5C is generated by largely the same process as that for Fig. 4C, but with some changes in parameters. The average signal $\overline{s_{ij}}$ is set to 25. The area of the kernel I is set to 0.05. In the creation of Fig. 5C, the signal timescale values are converted into units of kernel timescale by multiplying by a factor of $\frac{1}{\tau_k} = 20$, and A_{min} is replaced by dynamic range $\frac{A_{max}}{A_{min}}$ for $A_{max} = 100$. Outliers in the data are removed (points with negative offset, or offset above 0.5), which reflect response curves not of the form analyzable by our offset calculating function, and therefore not suited to approximating contrastive learning with the present scheme. The colorbar is normalized to span the range of values of $\log_{10}(\frac{\tau_f}{\tau_k})$. A 1000 point scatterplot is made for offset vs τ_f.

C. Markov kernel data

The final batch of data, used for Fig. 6B, randomly generates kernels using Markov chains and tests them on an otherwise fixed protocol. A total of 5,000 random kernels are generated. The tuneable parameters in the creation of each kernel are F, γ, and Δs_{ij}. F and γ are defined in Section S8, and Δs_{ij} is the height of the step function passed as

signal. For every 50 kernels, random values of gamma (10 of order unity and 40 ranging from order 1 to order 10^{-25}) are generated, and Δs_{ij} is chosen randomly from (5, 250) with the randint function from NumPy random library. The rate parameter F is chosen randomly from (1, 100). Each kernel is generated following the process outlined in Section S8. Once the kernels are created, they are normalized to have first moment 1 and their areas are computed.

For each kernel, a weight update curve is generated with the parameters ($A_{max} = 50, \tau_f = 3, \tau_s = 15, \overline{s_{ij}} = 10$) and θ_g is chosen using the rule of Eq. S48. The kernel, its randomly generated values of γ, Δs, F, its area, and the lists of x and y values that make up the response curve are all saved into a NumPy array.

Since the randomly generated Markov kernels have much less well defined and consistent shapes than those generated with low area *E. coli* kernels (the new kernels have area ranging from 0 to order 1), we use a different method of calculating offset and dynamic range. Rather than being distinctly split into three mostly linear regions, the resulting response curves are much more curved. To address this, we defined A_{min} as the minimum amplitude value for which the response curve crossed a threshold value of 0.05. Since the response curves are less linear than those of the low area *E. coli* curves, the offset was redefined here to be the y-intercept of the line of best fit for the data points between A_{min} and A_{max}. Next dissipation is calculated using Eq. S62. Then the inverse of dynamic range, offset and dissipation for each protocol are saved to a NumPy array of length 5000.

[1] S.-i. Yi, J. D. Kendall, R. S. Williams, and S. Kumar, Nature Electronics **6**, 45 (2023).
[2] J. Laydevant, D. Marković, and J. Grollier, Nature Communications **15**, 3671 (2024).
[3] S. Dillavou, M. Stern, A. J. Liu, and D. J. Durian, Physical Review Applied **18**, 014040 (2022).
[4] S. Dillavou, B. D. Beyer, M. Stern, M. Z. Miskin, A. J. Liu, and D. J. Durian, arXiv preprint arXiv:2311.00537 (2023).
[5] J. F. Wycoff, S. Dillavou, M. Stern, A. J. Liu, and D. J. Durian, The Journal of Chemical Physics **156** (2022).
[6] E. Williams, C. Bredenberg, and G. Lajoie, in *International Conference on Machine Learning* (PMLR, 2023) pp. 37042–37065.
[7] M. Ernoult, J. Grollier, D. Querlioz, Y. Bengio, and B. Scellier, arXiv preprint arXiv:2005.04168 (2020).
[8] P. Baldi and F. Pineda, Neural Computation **3**, 526 (1991), https://direct.mit.edu/neco/article-pdf/3/4/526/812186/neco.1991.3.4.526.pdf.
[9] V. R. Anisetti, A. Kandala, B. Scellier, and J. Schwarz, Neural Computation , 1 (2024).
[10] G.-q. Bi and M.-m. Poo, Annual review of neuroscience **24**, 139 (2001).
[11] A. N. Burkitt, H. Meffin, and D. B. Grayden, Neural Computation **16**, 885 (2004).
[12] T. Moraitis, A. Sebastian, and E. Eleftheriou, arXiv preprint arXiv:2009.06808 (2020).
[13] X. Xie and H. S. Seung, Advances in neural information processing systems **12** (1999).
[14] E. Martin, M. Ernoult, J. Laydevant, S. Li, D. Querlioz, T. Petrisor, and J. Grollier, Iscience **24** (2021).
[15] A. Journé, H. G. Rodriguez, Q. Guo, and T. Moraitis, arXiv preprint arXiv:2209.11883 (2022).
[16] D. Krotov and J. J. Hopfield, Proceedings of the National Academy of Sciences **116**, 7723 (2019).
[17] B. Scellier, S. Mishra, Y. Bengio, and Y. Ollivier, arXiv preprint arXiv:2205.15021 (2022).
[18] G. Hinton, arXiv preprint arXiv:2212.13345 (2022).
[19] B. Scellier and Y. Bengio, Frontiers in computational neuroscience **11**, 24 (2017).
[20] S. Dillavou, B. D. Beyer, M. Stern, A. J. Liu, M. Z. Miskin, and D. J. Durian, Proceedings of the National Academy of Sciences **121**, e2319718121 (2024).
[21] C. H. Bennett, Studies In History and Philosophy of Science Part B: Studies In History and Philosophy of Modern Physics **34**, 501 (2003).
[22] J. Kendall, R. Pantone, K. Manickavasagam, Y. Bengio, and B. Scellier, arXiv preprint arXiv:2006.01981 (2020).
[23] X. Glorot and Y. Bengio, in *Proceedings of the thirteenth international conference on artificial intelligence and statistics* (JMLR Workshop and Conference Proceedings, 2010) pp. 249–256.
[24] A. Celani and M. Vergassola, Proceedings of the National Academy of Sciences **107**, 1391 (2010).
[25] G. Lan, P. Sartori, S. Neumann, V. Sourjik, and Y. Tu, Nature physics **8**, 422 (2012).
